# 

**Published:** 1879-02

**Authors:** 


					﻿FEBRUARY, 1879.
TWENTY-SIXTH YEAR—No 302.
HALL’S
Journal of Health.
PUBLISHED AT 349 SIXTH AVENUE,
(Near 22d Street),
NEW YORK.
E. H. GIBBS, A.M., M.D., Editor.
AGENTS FOR THE TRADE:
AMERICAN NEWS COMPANY AND NEW YORK NEWS COMPANY
Terms, $1.50 a Year, or $1.00 for Eight Months. Postage paid.
SINGLE NUMBER, 15 CENTS.
Couch & Johnston, Printers, 11 Frankfort Street, New York.
				

